# The clinical impacts and risk factors for non-central line-associated bloodstream infection in 5046 intensive care unit patients: an observational study based on electronic medical records

**DOI:** 10.1186/s13054-019-2353-5

**Published:** 2019-02-18

**Authors:** Shichao Zhu, Yan Kang, Wen Wang, Lin Cai, Xin Sun, Zhiyong Zong

**Affiliations:** 1 Department of Infection Control, West China Hospital of Sichuan University, Chengdu, China; 2 Intensive Care Unit, West China Hospital of Sichuan University, Chengdu, China; 3 Chinese Evidence-based Medicine Centre and CREAT Group, State Key Laboratory of Biotherapy, West China Hospital, Sichuan University and Collaborative Innovation Centre, Chengdu, 610041 Sichuan China; 4 Center of Infectious Diseases, West China Hospital of Sichuan University, Chengdu, 610041 Sichuan China

**Keywords:** Healthcare-associated infection, Bloodstream infection, Non-central line-associated bloodstream infection, Clinical impact, Risk factor

## Abstract

**Background:**

Most of the previous studies focused on central line-associated bloodstream infection (CLABSI), while non-central line-associated bloodstream infection (N-CLABSI) was poorly studied. This study was performed to investigate the clinical impacts and risk factors for N-CLABSI in intensive care unit (ICU) patients.

**Methods:**

An observational study was conducted in an adult general ICU. The electronic medical records from 2013 to 2017 of all patients aged ≥ 18 years admitted to the ICU > 2 days were analyzed retrospectively. Patients with N-CLABSI and without N-CLABSI or with CLABSI were compared for clinical features and outcomes. Predicted death in ICU included death in ICU and discharging from ICU against medical advice because of critical conditions and the desire to pass away at home. Propensity score (PS) matching was used to ensure that both two groups had similar baseline characteristics. Multivariate regression models were used to confirm whether N-CLABSI was an independent risk factor for each of the outcomes and to analyze the risk factors for N-CLABSI in ICU patients.

**Results:**

Of 5046 patients included, 155 developed 168 ICU-acquired N-CLABSI episodes (2.1 episodes per 1000 patient-days) in the ICU, accounted for the majority of nosocomial bloodstream infections (NBSIs; 71.8%). After PS matching, patients with N-CLABSI had prolonged length of stay (LOS) in ICU (median 15 days, *p* <  0.001) and LOS in hospital (median 13 days, *p* <  0.001), excess hospitalization costs (median, $27,668 [in US dollar 2017, 1:6.75], *p* <  0.001), and increased mortality in ICU (8.8%, *p* = 0.013) and predicted mortality in ICU (22.7%, *p* <  0.001), compared with those without N-CLABSI. There were no significant differences in all the outcomes between N-CLABSI and CLABSI. N-CLABSI was an independent risk factor for each of the outcomes. Gastrointestinal bleeding (adjusted odds ratio [aOR] 2.30), trauma (aOR 2.52), pancreatitis (aOR 3.45), surgical operation (aOR 1.82), intravascular catheters (aOR 2.93), sepsis (aOR 1.69), pneumonia (aOR 1.53), intraabdominal infection (IAI, aOR 8.37), or healthcare-associated infections other than NBSI, pneumonia, and IAI (aOR 3.89) were risk factors for N-CLABSI in ICU patients.

**Conclusions:**

N-CLABSI was associated with similar poor outcomes with CLABSI, including prolonged LOS in ICU and in hospital and increased hospitalization costs and predicted mortality in ICU. The risk factors for N-CLABSI identified in this study provide further insight in preventing N-CLABSI.

**Electronic supplementary material:**

The online version of this article (10.1186/s13054-019-2353-5) contains supplementary material, which is available to authorized users.

## Background

Nosocomial bloodstream infections (NBSIs) are one of the most severe types of healthcare-associated infections (HAIs), are a major cause of mortality, and are associated with significantly prolonged length of stay (LOS) in hospital and increased healthcare costs [1–3], especially in intensive care units (ICUs) [4]. NBSIs are consisted of central line-associated bloodstream infections (CLABSIs) and non-central line-associated bloodstream infections (N-CLABSIs) according to the American Centers for Disease Control and Prevention (CDC)-National Healthcare Safety Network (NHSN) [5]. Most of the previous studies focused on CLABSIs, which have clear preventability. Several evidence-based guidelines have been developed to prevent and reduce CLABSIs [6, 7]. A 46% reduction in CLABSIs has been obtained in hospitals across the USA from 2008 to 2013 [8].

By contrast, N-CLABSI is a forgotten issue that has been poorly studied, especially in the Chinese population. N-CLABSI may be more common in NBSIs along with the reduction of CLABSI. It is still uncertain about whether patients with N-CLABSI have poorer outcomes than those without N-CLABSI or with CLABSI and whether N-CLABSI could be prevented. This study was performed to investigate the clinical impacts and risk factors for N-CLABSI in ICU patients to identify risk factors that could be controlled.

## Methods

### Study design and settings

This observational study was conducted using electronic medical record (EMR) data in a general ICU of West China Hospital, Sichuan University, southwestern China. It is a 50-bed ICU for adult patients that receives approximately 1300 admissions per year, in a 5000-bed tertiary-care hospital. It is the major referral center in western China for patients with a variety of critical conditions such as severe acute pancreatitis, malignancy, liver transplantation, multiple organ failure, and severe pneumonia.

### Study population

All patients aged ≥ 18 years admitted to the ICU > 2 days from January 2013 to December 2017 were eligible for inclusion in this study. Those with long ICU stay because of medical disputes or with both N-CLABSI and CLABSI during the same ICU stay were excluded. The exposed group in the cohort for the outcomes was consisted of all the patients with ICU-acquired N-CLABSI, while the control group consisted of all the patients without ICU-acquired N-CLABSI (including those with CLABSI).

### Definitions

N-CLABSIs in this study were consisted of primary and secondary BSIs defined by the American CDC-NHSN [5]. A N-CLABSI developed 2 days after admission to the ICU and within 2 days of discharge from the ICU was defined as an ICU-acquired N-CLABSI. BSI date was defined as the date of the positive blood sampling. A BSI that occurred after the NHSN 14-day repeat infection timeframe of the previous BSI was defined as a new BSI. For the analysis of clinical impacts, the confounders were considered during the whole stay in ICU. For the analysis of risk factors, possible factors were considered prior to the N-CLABSI date in patients with N-CLABSI, while those were considered during the whole stay in ICU in patients without N-CLABSI. For patients with 2 or more N-CLABSI episodes, possible factors were analyzed prior to the first N-CLABSI episode.

### Outcomes

The outcomes in this study were consisted of LOS in ICU, LOS in hospital, hospitalization costs, death in ICU (all-cause mortality), and predicted death in ICU. Predicted death in ICU included death in ICU and discharging from ICU against medical advice because of critical conditions and the desire to pass away at home.

### Data source

All patient data were retrieved from the medical record database of the EMR system, which was established in 2009, with annual discharged patients exceeding 200,000. Prospective surveillance of HAIs was performed by infection control practitioners and ICU nurses, using the HAI definitions updated annually by the American CDC-NHSN [5]. This study was approved (No. WCH2018-409) by the Ethics Committee of West China Hospital, Sichuan University, with the waiver of informed consents. Patient data were anonymized prior to analysis.

### Data collection

Patient data included demographic characteristics (age, sex), illness severity, comorbidities on ICU admission, invasive procedures, multidrug-resistant organisms (MDROs), HAIs, and outcomes. Illness severity was evaluated by Acute Physiology and Chronic Health Evaluation (APACHE) II score [9] during the first 24 h on ICU admission. Comorbidities on ICU admission including chronic underlying diseases (diabetes, hypertension, respiratory diseases, cardiovascular diseases, renal diseases, liver diseases, and malignancies), disorder of consciousness, gastrointestinal bleeding, trauma, immunological diseases, organ transplantation, multiple organ failure, shock, pancreatitis, acute respiratory distress syndrome (ARDS), and sepsis were retrieved from the diagnoses on ICU admission. Invasive procedures included surgical operation, intravascular catheters, organ biopsy, mechanical ventilation (MV), and urinary catheters (UC). All invasive procedures were conducted after hospital admission according to the medical orders in the EMR system, except surgical operation. Surgical operation and MDROs were considered from 1 year prior to this ICU admission. HAIs including BSI were retrieved from HAI surveillance. Pneumonia in the comorbidities on ICU admission and in HAIs was combined for analyzing, as well as intraabdominal infection (IAI). The outcomes were retrieved or evaluated from the medical records (predicted deaths were evaluated from the discharge records of EMR).

### Statistical analysis and bias control

Statistical analysis was performed using the SPSS program (version 19.0; SPSS, Chicago, IL, USA) and the STATA software (version 15.0; StataCorp LLC, USA). Continuous numerical variables were described as means and standard deviations (SD) when fitting a normal distribution, otherwise as medians and interquartile ranges. The independent sample *T* test was used to determine the differences in means after the homogeneity test of variances, while the Wilcoxon signed rank tests for median values. Categorical data were analyzed by the chi-square test or continuous correction chi-square test. Propensity score (PS) matching was used to ensure that both the two groups had similar baseline characteristics in the cohorts of the outcomes, which was estimated applying multivariable logistic regression models. A standardized difference ≤ 0.1 for a baseline covariate reveals a negligible imbalance [10]. The clinical impacts were analyzed by the new cohorts after PS matching, by multiple linear regression models (non-normally distributed outcomes were conducted by logarithmic transformations) or logistic regression models. To ensure the parsimony of the final multivariate regression models for the outcomes and N-CLABSI, variables for inclusion were carefully chosen, given the number of cases available. The variables with a *p* value < 0.1 in the univariate analysis and those that were considered clinically relevant according to the advices of clinical experts and previous studies were selected into the multivariate regression models. Multicollinearity was analyzed for all the independent variables in the final models. The interaction between the factors was investigated. All tests were two-sided, and *p* <  0.05 was considered statistically significant.

## Results

### Epidemiology of N-CLABSI in ICU patients

**Fig. 1:**
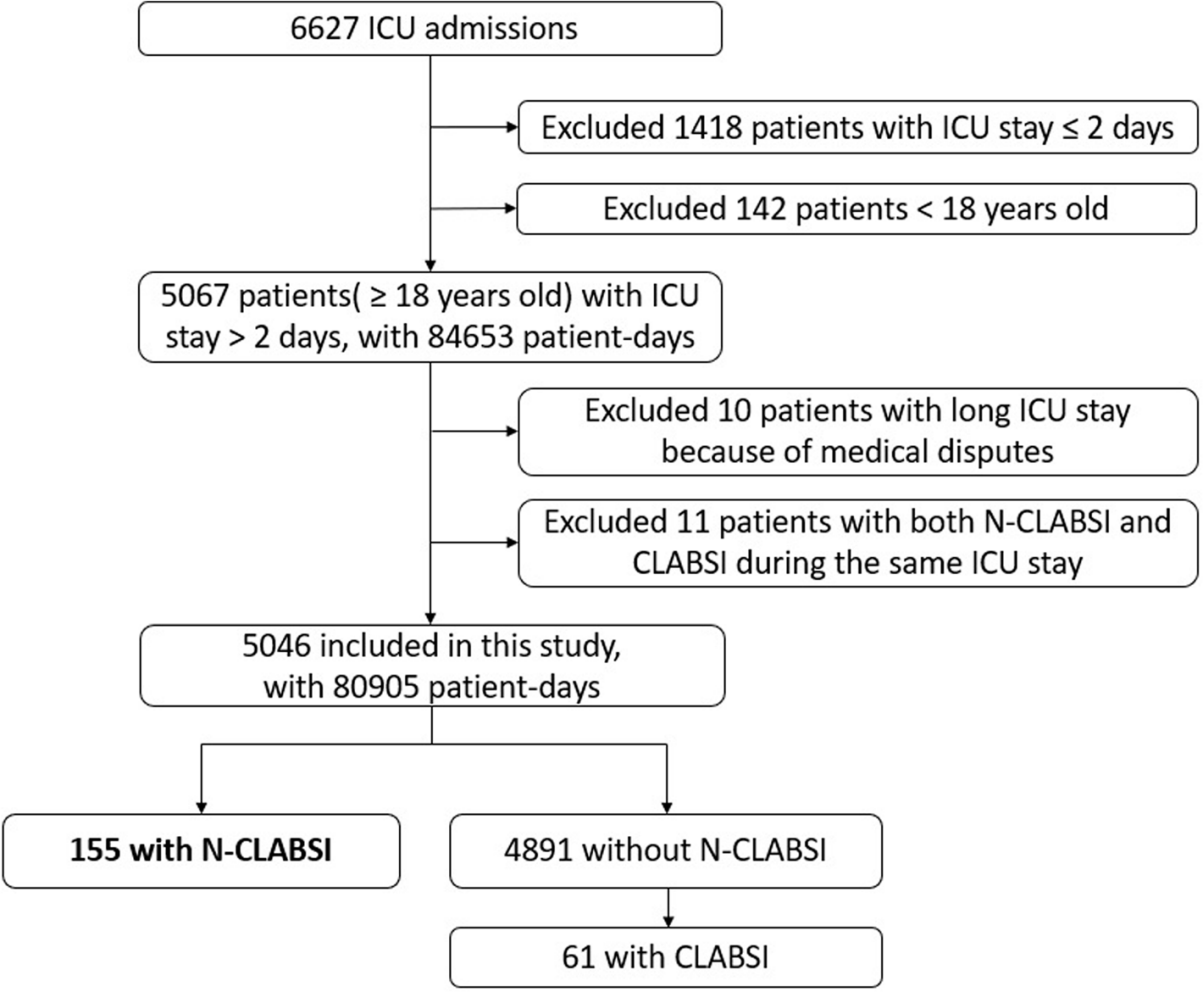
Inclusion and exclusion of patients in this study. ICU, intensive care unit; N-CLABSI, non-central line-associated bloodstream infection; CLABSI, central line-associated bloodstream infection

**Fig. 2:**
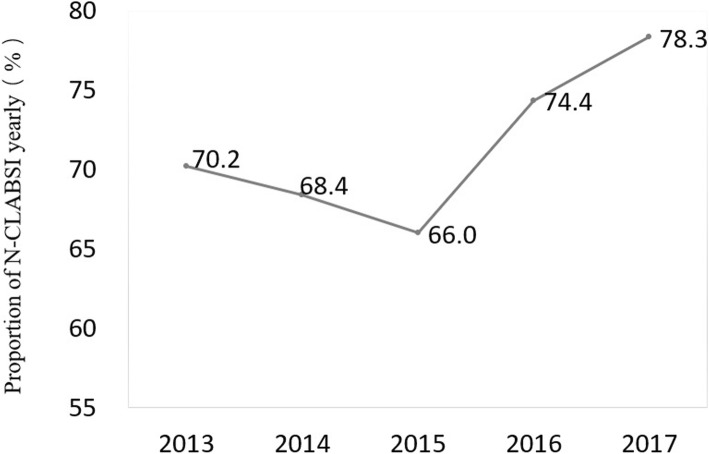
Proportion of N-CLABSI in NBSIs yearly. N-CLABSI, non-central line-associated bloodstream infection; NBSIs, nosocomial bloodstream infections

A total of 6627 patients with 87,984 patient-days (13.3 days for the average LOS in ICU) were admitted to the ICU during this 5-year period. As shown in Fig. 1, 1,418 patients with LOS in ICU ≤ 2 days, 142 aged < 18 years, 10 with long ICU stay because of medical disputes, and 11 with both N-CLABSI and CLABSI during the same ICU stay were excluded. After the exclusion of non-eligible patients, 5046 patients with 80,905 patient-days were included in this study, of which 155 (3.1 cases per 100 patients, 1.9 cases per 1000 patient-days) developed 168 ICU-acquired N-CLABSI episodes (2.1 episodes per 1000 patient-days) in the ICU (Fig. 1). Patients with N-CLABSI had a much higher proportion (71.8%) in the patients with NBSI than those with CLABSI (61 cases, 28.2%). The proportion of N-CLABSI increased from 70.2% in 2013 to 78.3% in 2017 (Fig. 2).

**Fig. 3:**
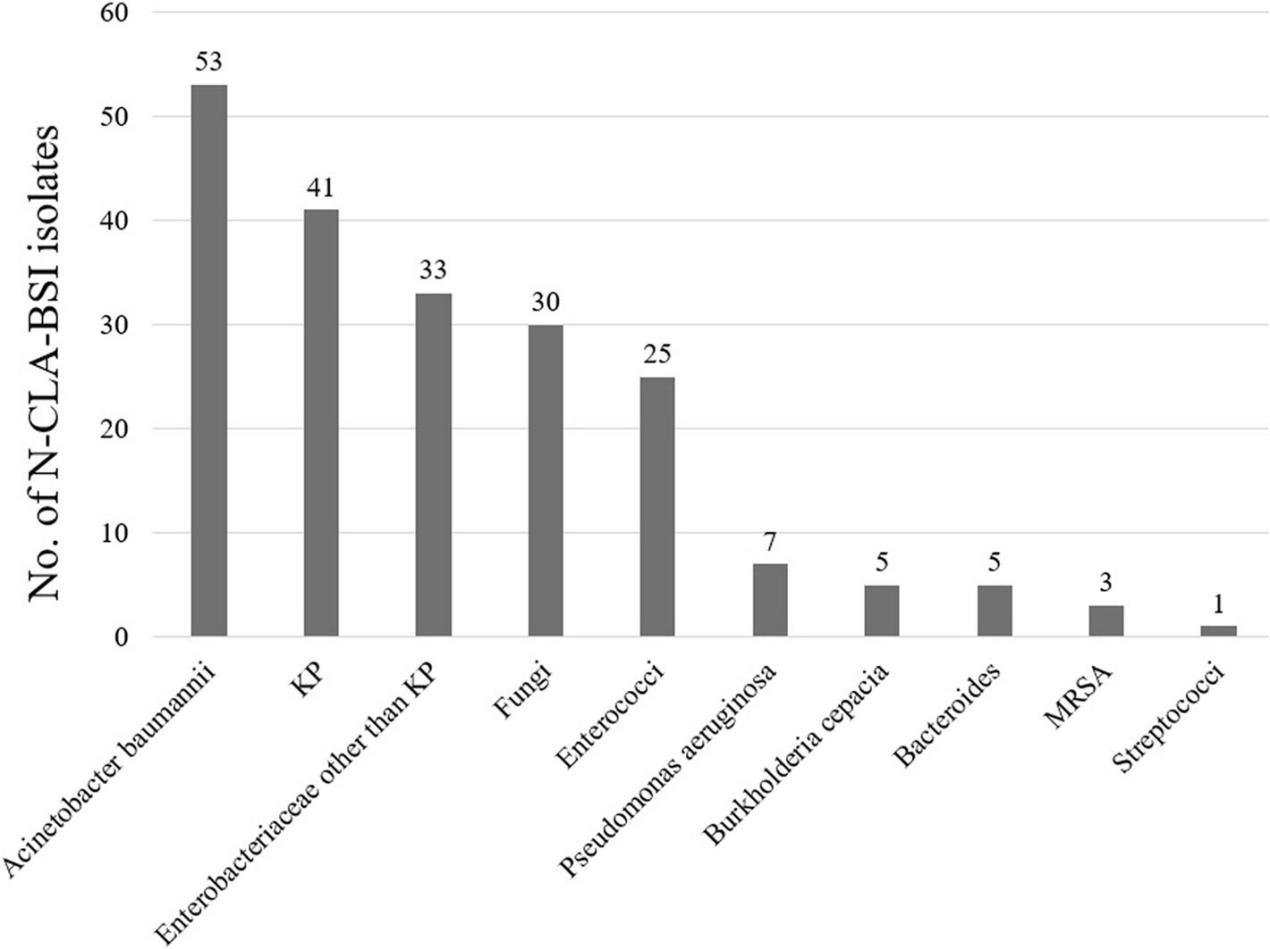
Frequency of isolates of N-CLABSI pathogens. N-CLABSI, non-central line-associated bloodstream infection; KP, *Klebsiella pneumoniae*; MRSA, methicillin-resistant *Staphylococcus aureus*

The 155 patients with N-CLABSI were 20 to 97 years old (mean, 53.3 years old), were mostly male (110, 71.0%), and had a mean APACHE II score on ICU admission of 21.0. N-CLABSI developed 3–116 days after admission to ICU (median, 13 days). Most N-CLABSIs (*n* = 135, 87.1%) were secondary BSIs, while the remaining 20 (12.9%) were primary BSIs. There were 203 bacterial or fungal strains recovered from the 168 N-CLABSI episodes. *Acinetobacter baumannii* (53 isolates, 26.1%) was the most common followed by *Klebsiella pneumoniae* (41 isolates, 20.2%) (Fig. 3).

### Comparisons of patients with N-CLABSI versus without N-CLABSI or with CLABSI

Baseline demographic and clinical features of all 5046 eligible patients included in this study and results of comparisons of patients with N-CLABSI versus those without N-CLABSI or with CLABSI are shown in Table 1. Patients with N-CLABSI have significantly longer LOS in ICU (median, 32 vs 10 days, by 22 days, *p* <  0.001), longer LOS in hospital (median, 42 vs 21 days, by 21 days, *p* <  0.001), higher hospitalization costs (median, 305,074 vs 97,591 yuan [difference 207,483-yuan, equivalent to USD 30,738], *p* <  0.001), increased mortality in ICU (25.2% vs 13.0%, by 12.2%, *p* <  0.001), and increased predicted mortality in ICU (58.1% vs 27.5%, by 20.6%, *p* <  0.001) than those without N-CLABSI. Interestingly, there were no significant differences in all the outcomes (additionally including LOS in ICU or hospital after NBSI date) between the patients with N-CLABSI and those with CLABSI (*p* > 0.05).

**Table 1:** Baseline clinical features and outcomes of patients with N-CLABSI or without N-CLABSI

Features	Patients with N-CLABSI (*n* = 155) (group A)	Patients without N-CLABSI (*n* = 4891) (group B*)	Patients with CLABSI (*n* = 64) (group B-sub)	*p* value^†^	
				A vs. B	A vs. B-sub
Age (years), mean ± SD	53.3 ± 16.4	56.9 ± 17.7	58.4 ± 16.0	*0.012*	*0.039*
Sex, no. male (%)	110 (71.0)	3174 (64.9)	43 (70.5)	0.118	0.945
APACHE II score on ICU admission, mean ± SD	21.0 ± 8.0	19.0 ± 8.1	22.4 ± 8.5	*0.002*	0.272
Chronic underlying diseases, no. (%)	107 (69.0)	3342 (68.3)	51 (83.6)	0.853	*0.030*
Diabetes, no. (%)	30 (19.4)	619 (12.7)	18 (29.5)		
Hypertension, no. (%)	35 (22.6)	1028 (21.0)	21 (34.4)		
Respiratory diseases, no. (%)	16 (10.3)	654 (13.4)	7 (11.5)		
Cardiovascular diseases, no. (%)	27 (17.4)	744 (15.2)	15 (24.6)		
Renal diseases, no. (%)	23 (14.8)	609 (12.5)	12 (19.7)		
Liver diseases, no. (%)	52 (33.5)	1147 (23.5)	26 (42.6)		
Malignancies, no. (%)	18 (11.6)	1138 (23.3)	10 (16.4)		
Disorder of consciousness, no. (%)	21 (13.5)	479 (9.8)	10 (16.4)	0.123	0.591
Gastrointestinal bleeding, no. (%)	25 (16.1)	395 (8.1)	9 (14.8)	*< 0.001*	0.803
Trauma, no. (%)	30 (19.4)	720 (14.7)	6 (9.8)	0.110	0.091
Immunological diseases, no. (%)	18 (11.6)	767 (15.7)	11 (18.0)	0.169	0.213
Organ transplantation, no. (%)	15 (9.7)	536 (11.0)	8 (13.1)	0.615	0.461
Multiple organ failure, no. (%)	62 (40.0)	1150 (23.5)	24 (39.3)	*< 0.001*	0.929
Shock, no. (%)	69 (44.5)	1118 (22.9)	25 (41.0)	*< 0.001*	0.637
Pancreatitis, no. (%)	70 (45.2)	597 (12.2)	30 (32.8)	*< 0.001*	0.097
ARDS, no. (%)	26 (16.8)	225 (4.6)	6 (9.8)	*< 0.001*	0.196
Surgical operation, no. (%)	120 (77.4)	3024 (61.8)	44 (72.1)	*< 0.001*	0.413
Intravascular catheters, no. (%)	140 (90.3)	2962 (60.6)	61 (100.0)	*< 0.001*	*0.012*
Organ biopsy, no. (%)	31 (3.7)	812 (16.6)	17 (27.9)	0.264	0.210
MV, no. (%)	141 (91.0)	4386 (89.7)	56 (91.8)	0.602	0.845
UC, no. (%)	148 (95.5)	4690 (95.9)	57 (93.4)	0.152	0.539
MDRO, no. (%)	67 (43.2)	1660 (33.9)	41 (67.2)	*0.017*	*0.002*
Sepsis, no. (%)	38 (24.5)	332 (6.8)	11 (18.0)	*< 0.001*	0.306
Pneumonia, no. (%)	108 (69.7)	2648 (54.1)	45 (73.8)	*< 0.001*	0.551
IAI, no. (%)	87 (56.1)	371 (7.6)	7 (11.5)	*< 0.001*	*< 0.001*
HAIs other than NBSIs, pneumonia and IAI, no. (%)	17 (11.0)	161 (3.3)	5 (8.2)	*< 0.001*	0.544
Days prior to NBSI date from ICU admission, median (IQR)	13.0 (8.0–25.0)	NA	19.0 (12.5–30.0)	NA	*0.031*
Outcomes					
LOS in ICU after NBSI date (days), median (IQR)	15.0 (8.0–27.0)	NA	16.0 (6.0–30.0)	NA	0.876
LOS in hospital after NBSI date (days), median (IQR)	18.0 (8.0–37.0)	NA	19.0 (9.5–36.5)	NA	0.729
LOS in ICU (days), median (IQR)	32.0 (18.0–55.0)	10.0 (5.0–18.0)	34.0 (20.0–59.5)	*< 0.001*	0.308
LOS in hospital (days), median (IQR)	42.0 (24.0–68.0)	21.0 (13.0–33.0)	39.0 (26.0–77.5)	*< 0.001*	0.533
Hospitalization costs (CNY), median (IQR)	305,074 (196,575–452,309)	97,591 (57,306–190,754)	35,9646 (229,092–549,737)	*< 0.001*	0.103
Deaths in ICU (mortality)	39 (25.2)	637 (13.0)	20 (32.8)	*< 0.001*	0.258
Predicted deaths in ICU (predicted mortality)	90 (58.1)	1343 (27.5)	35 (57.4)	*< 0.001*	0.927

Predicted deaths in ICU included deaths in ICU and patients discharged from ICU against medical advice because of critical conditions and the desire to pass away at home

*N-CLABSI*, non-central line-associated bloodstream infection; *SD*, standard deviation; *APACHE*, Acute Physiology and Chronic Health Evaluation; *ICU*, intensive care unit; *ARDS*, acute respiratory distress syndrome; *MV*, mechanical ventilation; *UC*, urinary catheter; *MDRO*, multidrug-resistant organism; *IAI*, intraabdominal infection; *HAI*, healthcare-associated infection; *NBSI*, nosocomial bloodstream infection; *IQR*, interquartile range; *CNY*, China Yuan; *LOS*, length of stay

*Group B included group B-sub

^†^*p* values < 0.05 are shown in italics

### Impacts of N-CLABSI on the outcomes

Three PS models (matching ratio 1:4, calipers value 0.02) were developed to control the confounders in the cohorts (one for logarithmic value of LOS in ICU and logarithmic value of LOS in hospital, one for logarithmic value of hospitalization costs, and another for death in ICU and predicted death in ICU). In all the three matched cohorts, the standardized differences were 0.1 or less for all baseline characteristics, demonstrating only minor differences between both the two groups (more details were shown in additional files (see Additional files 1, 2, and 3)). As shown in Table 2, patients with N-CLABSI did have prolonged LOS in ICU (median 15.0 days, *p* <  0.001) and LOS in hospital (median 13.0 days, *p* <  0.001), increased hospitalization costs (median, 186,757-yuan, equivalent to USD 27,668, *p* <  0.001), and excess mortality in ICU (8.8%, *p* = 0.013) and predicted mortality in ICU (22.7%, *p* <  0.001), compared with those without N-CLABSI. According to the results of the multivariate regression analysis in the PS-matched cohorts shown in Table 3, N-CLABSI was an independent risk factor for each of the outcomes respectively (the adjusting factors for the PS-matched cohorts were shown in an additional file (see Additional file 4)).

**Table 2:** Excess outcomes of N-CLABSI after PS matching

Outcomes	Matching ratio	With N-CLABSI	Without N-CLABSI	Excess values	*p* value^*^
LOS in ICU (days), median (IQR)	155:518	32.0 (18.0–55.0)	17.0 (9.0–31.0)	15.0	*< 0.001*
LOS in hospital (days), median (IQR)	155:518	42.0 (24.0–68.0)	29.0 (16.0–48.0)	13.0	*< 0.001*
Hospitalization costs (CNY), median (IQR)	155:525	305,074 (196,575–452,309)	118,317 (68,819–220,763)	186,757	*< 0.001*
Deaths in ICU (mortality)	153:487	38 (24.8%)	78 (16.0%)	8.8%	*0.013*
Predicted deaths in ICU (predicted mortality)	153:487	89 (58.2%)	173 (35.5%)	22.7%	*< 0.001*

Predicted deaths in ICU included deaths in ICU and patients discharged from ICU against medical advice because of critical conditions and the desire to pass away at home

*N-CLABSI*, non-central line-associated bloodstream infection; *PS*, propensity score; *LOS*, length of stay; *ICU*, intensive care unit; *IQR*, interquartile range; *CNY*, China Yuan

*p values < 0.05 are shown in italics

**Table 3:** Relationships between N-CLABSI and each of the outcomes from multivariate analysis in the PS-matched cohorts

Outcomes	Adjusted β^*^ or OR^†^	95% CI	*p* value^‡^
Logarithmic value of LOS in ICU	*β* = 0.217	0.156–0.277	*< 0.001*
Logarithmic value of LOS in hospital	*β* = 0.137	0.076–0.197	*< 0.001*
Logarithmic value of hospitalization costs	*β* = 0.213	0.152–0.275	*< 0.001*
Death in ICU	OR = 2.175	1.294–3.656	*0.003*
Predicted death in ICU	OR = 2.960	1.850–4.737	*0.001*

Predicted death in ICU included death in ICU and discharging from ICU against medical advice because of critical conditions and the desire to pass away at home

*N-CLABSI*, non-central line-associated bloodstream infection; *PS*, propensity score; *β*, unstandardized coefficients of multiple linear regression model; *OR*, odds ratio; *CI*, confidence interval; *LOS*, length of stay; *ICU*, intensive care unit

*Continuous outcomes were analyzed by multiple linear regression models

^†^Categorical outcomes were analyzed by logistic regression models

^‡^*p* values < 0.05 are shown in italics

### Risk factors for N-CLABSI in ICU patients

As shown in Table 4, the variables with *p* <  0.1 in the univariate logistic regression models and those that were considered clinically relevant were selected into the multivariate logistic regression model for N-CLABSI. There was no multicollinearity within the variables in the final model. Patients with gastrointestinal bleeding (aOR 2.30, 95% CI 1.38–3.82, *p* = 0.001), trauma (aOR 2.52, 95% CI 1.57–4.04, *p* <  0.001), pancreatitis (aOR 3.45, 95% CI 2.24–5.30, *p* <  0.001), surgical operation (aOR 1.82, 95% CI 1.19–2.78, *p* = 0.006), intravascular catheters (aOR 2.93, 95% CI 1.65–5.22, *p* <  0.001), sepsis (aOR 1.69, 95% CI 1.09–2.63, *p* = 0.02), pneumonia (aOR 1.53, 95% CI 1.03–2.28, *p* = 0.038), IAI (aOR 8.37, 95% CI 5.63–12.44, *p* <  0.001), or HAIs other than NBSI, pneumonia, and IAI (aOR 3.89, 95% CI 2.09–7.24, *p* <  0.001) were at higher risk for developing N-CLABSI in ICU patients.

**Table 4:** Risk factors for N-CLABSI in ICU patients (logistic regression)

Variables*	Univariate analysis		Multivariate analysis	
	OR (95% CI)	*p* value^†^	Adjusted OR (95% CI)	*p* value^‡^
*Age (years)*	*0.988 (0.980–0.997)*	*0.012*	1.004 (0.993–1.016)	0.431
Sex (male)	1.322 (0.930–1.880)	0.120		
*APACHE II score on ICU admission*	*1.032 (1.010–1.050)*	*0.002*	1.010 (0.986–1.034)	0.411
Chronic underlying diseases	1.033 (0.731–1.460)	0.853		
Disorder of consciousness	1.443 (0.903–2.309)	0.125		
*Gastrointestinal bleeding*	*2.189 (1.409–3.399)*	*< 0.001*	*2.295 (1.377–3.824)*	*0.001*
*Trauma*	1.390 (0.926–2.087)	0.112	*2.521 (1.573–4.040)*	*< 0.001*
Immunological diseases	0.706 (0.430–1.160)	0.171		
Organ transplantation	0.871 (0.507–1.494)	0.615		
*Multiple organ failure*	*2.169 (1.562–3.011)*	*< 0.001*	1.093 (0.734–1.627)	0.663
*Shock*	*2.708 (1.959–3.743)*	*< 0.001*	1.005 (0.683–1.479)	0.979
*Pancreatitis*	*5.923 (4.268–8.220)*	*< 0.001*	*3.445 (2.239–5.300)*	*< 0.001*
*ARDS*	*4.180 (2.686–6.503)*	*< 0.001*	1.673 (0.982–2.850)	0.058
*Surgical operation*	*2.117 (1.446–3.098)*	*< 0.001*	*1.815 (1.186–2.776)*	*0.006*
*Intravascular catheters*	*6.078 (3.558–10.384)*	*< 0.001*	*2.934 (1.649–5.221)*	*< 0.001*
Organ biopsy	1.256 (0.841–1.875)	0.265		
MV	1.160 (0.664–2.024)	0.602		
UC	0.621 (0.322–1.198)	0.155		
*MDRO positive*	*1.481 (1.072–2.046)*	*0.017*	0.732 (0.500–1.069)	0.107
*Sepsis*	*4.460 (3.042–6.538)*	*< 0.001*	*1.693 (1.088–2.633)*	*0.020*
*Pneumonia*	*1.946 (1.376–2.754)*	*< 0.001*	*1.528 (1.025–2.277)*	*0.038*
*IAI*	*15.185 (10.873–21.206)*	*< 0.001*	*8.369 (5.630–12.440)*	*< 0.001*
*HAIs other than NBSI, pneumonia and IAI*	*3.619 (2.135–6.135)*	*< 0.001*	*3.888 (2.087–7.244)*	*< 0.001*

*BSI*, bloodstream infection; *N-CLABSI*, non-central line-associated bloodstream infection; *ICU*, intensive care unit; *OR*, odds ratio; *APACHE*, Acute Physiology and Chronic Health Evaluation; *ARDS*, acute respiratory distress syndrome; *MV*, mechanical ventilation; *UC*, urinary catheter; *MDRO*, multidrug-resistant organism; *IAI*, intraabdominal infection; *HAI*, healthcare-associated infection

*The variables selected into the multivariate logistic regression model are shown in italics

^†^Variables were analyzed by univariate logistic regression models. Parameters with *p* < 0.1 are shown in italics

^‡^Variables were analyzed by a multiple logistic regression model. Parameters with statistical significance (*p* < 0.05) are shown in italics

## Discussion

To our knowledge, this is the first time to identify the clinical impacts and risk factors for N-CLABSI in ICU patients. This study has several notable findings including (1) most of the NBSIs were N-CLABSIs in our ICU; (2) N-CLABSI was associated with poor outcomes, which include prolonged LOS in ICU and in hospital and increased hospitalization costs and all-cause mortality in ICU; (3) N-CLABSI had similar clinical impacts with CLABSI; and (4) the risk factors for N-CLABSI in this study provide possibilities to prevent N-CLABSI.

There are very few studies that reported the incidence of N-CLABSIs in NBSIs and in ICU patients directly. The proportion of N-CLABSIs could be calculated in the studies focusing on CLABSIs. The high proportion (71.8%) of N-CLABSIs in NBSIs of our study is consistent with that in the study of Laura et al. (78.0% in 2009, not shown in the study directly) [11] while is much higher than 37.6% (50/133) in the study of Jinhong [12] and 36.0% (44/122) in the study of Christopher et al. [13]. Furthermore, the high incidence of N-CLABSIs in this study (2.3 cases per 100 ICU admissions) approximates to the total incidence of NBSIs (2.67 cases per 100 ICU admissions) in the previous study [1].

Previous studies have identified that both NBSIs (as a whole) and CLABSIs were associated with poor clinical outcomes, including LOS in hospital, healthcare costs, and mortality. The prolonged LOS in ICU of CLABSIs varied from 10 to 20 days [1, 14–17], excess costs of CLABSIs from $3700 to $39,000 [14, 18–21], and the increased mortality of CLABSIs from 4 to 37% [1, 16, 22, 23]. These ranges contained the excess outcomes of N-CLABSIs in this study. However, few studies have estimated the difference in the clinical outcomes between N-CLABSIs and CLABSIs. Our study showed that N-CLABSIs had similar poor outcomes with CLABSIs. N-CLABSI was indeed a significant independent risk factor for each of the outcomes of ICU patients according to this study. Therefore, N-CLABSI should be paid more attention to than previous, especially in ICU patients.

Most previous studies focus on CLABSI with a probable reason that CLABSI has better preventability. However, the preventability of N-CLABSI is still uncertain. The risk factors for N-CLABSI in this study were significantly different from those for CLABSI [24–28]. These factors provide possibilities to prevent N-CLABSIs. Measures that could prevent pathogens from entering the bloodstream directly from the wounds or could control or prevent the infectious factors could be possible prevention strategies for N-CLABSI. For example, timely hemostasis for gastrointestinal bleeding; aseptic techniques for wound management, operation, and intravascular catheters; treating the primary infections; and preventing HAIs could be effective for preventing N-CLABSI in ICU patients. IAI (including ICU-acquired IAI) with aOR 8.37 and HAIs other than BSI, pneumonia, and IAI with aOR 3.89 were the strongest two independent risk factors for N-CLABSI. This suggests more necessity to prevent HAIs.

We are aware of the several limitations of this study. First, the relative frequency of N-CLABSIs that could be prevented was not evaluated in this study because it was difficult to identify the cause of a primary BSI and how many secondary BSIs could be prevented after primary site infections. Second, we just evaluated the medical data in one of the eight different types of ICUs of our hospital—a single-center design. The generalizability of our findings could be limited by the type of this ICU (a general ICU always admits critically ill patients) and the high prevalence of NBSIs in this unit.

## Conclusions

In conclusion, N-CLABSI had an increased high proportion in NBSIs and high prevalence in ICU patients. N-CLABSI was associated with poor clinical outcomes, which included LOS in ICU and in hospital, hospitalization costs, and all-cause mortality in ICU, like CLABSI. Gastrointestinal bleeding, trauma, pancreatitis, surgical operation, intravascular catheters, sepsis, pneumonia, IAI, and HAIs other than NBSI, pneumonia, and IAI were risk factors for developing N-CLABSI in ICU patients, which provided some possible measures for preventing N-CLABSI, such as aseptic techniques, treating the primary infections, and preventing other HAIs.

## Additional files

Additional file 1: **Table S1.** PS model for LOS outcomes. (DOCX 21 kb)

Additional file 2: **Table S2.** PS model for hospitalization costs. (DOCX 21 kb)

Additional file 3: **Table S3.** PS model for mortality. (DOCX 21 kb)

Additional file 4: **Table S4.** Adjusting factors for the clinical impacts of N-CLABSI in the PS-matched cohorts. (DOCX 18 kb)

## Abbreviations

aOR: Adjusted OR
APACHE: Acute Physiology and Chronic Health Evaluation
ARDS: Acute respiratory distress syndrome
BSI: Bloodstream infection
CDC: Centers for Disease Control and Prevention
CI: Confidence interval
CLABSI: Central line-associated bloodstream infection
CNY: China Yuan
EMR: Electronic medical record
HAI: Healthcare-associated infection
IAI: Intraabdominal infection
ICU: Intensive care unit
IQR: Interquartile range
KP: *Klebsiella pneumoniae*
LOS: Length of stay
MDRO: Multidrug-resistant organism
MRSA: Methicillin-resistant *Staphylococcus aureus*
MV: Mechanical ventilation
NBSI: Nosocomial BSI
N-CLABSI: Non-central line-associated bloodstream infection
NHSN: National Healthcare Safety Network
OR: Odds ratio
PS: Propensity score
SD: Standard deviation
UC: Urinary catheter
