# Supplementary material for: The clinical impacts and risk factors for non-central line-associated bloodstream infection in 5046 intensive care unit patients: an observational study based on electronic medical records
Source: Crit Care. 2019 Feb 18;23:52. doi: 10.1186/s13054-019-2353-5 (PMC6379966; doi:10.1186/s13054-019-2353-5)
Supplement: Supplementary file 1 — Table S1. PS model for LOS outcomes. (DOCX 21 kb) [file 13054_2019_2353_MOESM1_ESM.docx]

**Additional file 1**

**Table S1. PS model for LOS outcomes**

| Covariates | Before PS matching | | | After PS matching^†^ | | |
| --- | --- | --- | --- | --- | --- | --- |
|  | **N-CLABSI (n=155)** | **Without N-CLABSI (n=4891)** | **Standardized**  **Difference^*^** | **N-CLABSI (n=155)** | **Without N-CLABSI (n=501)** | **Standardized**  **Difference** |
| Age (y), mean ± SD | 53.3 ± 16.4 | 56.9 ± 17.7 | -0.213 | 53.3 ± 16.4 | 52.9 ± 18.1 | 0.024 |
| APACHE II score on ICU admission, mean ± SD | 21.0 ± 8.0 | 19.0 ± 8.1 | 0.249 | 21.0 ± 8.0 | 21.1 ± 7.6 | -0.002 |
| Gastrointestinal bleeding, No. (%) | 25 (16.1) | 395 (8.1) | 0.249 | 25 (16.1) | 73 (14.6) | 0.043 |
| Multiple organ failure, No. (%) | 62 (40.0) | 1,150 (23.5) | 0.360 | 62 (40.0) | 178 (35.5) | 0.092 |
| Disorder of consciousness, No. (%) | 21 (13.5) | 479 (9.8) | 0.117 | 21 (13.5) | 61 (12.2) | 0.041 |
| Trauma, No. (%) | 30 (19.4) | 720 (14.7) | 0.123 | 30 (19.4) | 109 (21.8) | 0.059 |
| Surgical operation, No. (%) | 138 (89.0) | 3,024 (61.8) | 0.639 | 138 (89.0) | 452 (87.3) | 0.100 |
| Intravascular catheters, No. (%) | 143 (92.3) | 2,962 (60.6) | 0.367 | 143 (92.3) | 458 (91.4) | 0.031 |
| Organ biopsy, No. (%) | 45 (29.0) | 812 (16.6) | 0.300 | 45 (29.0) | 125 (25.0) | 0.092 |
| MDRO, No. (%) | 123 (79.4) | 1,660 (33.9) | 1.031 | 123 (79.4) | 381 (76.0) | 0.080 |
| Pneumonia, No. (%) | 108 (69.7) | 2,648 (54.1) | 0.339 | 108 (69.7) | 338 (67.5) | 0.062 |

Note. PS, propensity score; LOS, length of stay; N-CLABSI, non-central line-associated bloodstream infection; SD, standard deviation; APACHE, Acute Physiology and Chronic Health Evaluation; ICU, intensive care unit; MDRO, multidrug-resistant organism.

^*^ An absolute value ≤ 0.1 indicates a negligible difference in the mean or prevalence of a covariate between groups.

^†^ Matching scale is 1:4, calipers value is 0.02.
