# Supplementary material for: The clinical impacts and risk factors for non-central line-associated bloodstream infection in 5046 intensive care unit patients: an observational study based on electronic medical records
Source: Crit Care. 2019 Feb 18;23:52. doi: 10.1186/s13054-019-2353-5 (PMC6379966; doi:10.1186/s13054-019-2353-5)
Supplement: Supplementary file 2 — Table S2. PS model for hospitalization costs. (DOCX 21 kb) [file 13054_2019_2353_MOESM2_ESM.docx]

**Additional file 2**

**Table S2. PS model for hospitalization costs**

| Covariates | Before PS matching | | | After PS matching^†^ | | |
| --- | --- | --- | --- | --- | --- | --- |
|  | **N-CLABSI (n=155)** | **Without N-CLABSI (n=4891)** | **Standardized**  **Difference^*^** | **N-CLABSI (n=155)** | **Without N-CLABSI (n=525)** | **Standardized**  **Difference** |
| Age (y), mean ± SD) | 53.3 ± 16.4 | 56.9 ± 17.7 | -0.213 | 53.3 ± 16.4 | 53.4 ± 17.5 | -0.006 |
| Sex, No. male (%) | 110 ± 71.0 | 3,174 ± 64.9 | 0.130 | 110 ± 71.0 | 377 ± 71.8 | -0.019 |
| APACHE II score on ICU admission, mean (SD) | 21.1 (8.0) | 19.0 (8.1) | 0.249 | 21.1 (8.0) | 20.8 (8.5) | 0.032 |
| Chronic underlying diseases, No. (%) | 107 (69.0) | 3,342 (68.3) | 0.117 | 107 (69.0) | 363 (69.1) | -0.002 |
| Trauma, No. (%) | 30 (19.4) | 720 (14.7) | 0.123 | 30 (19.4) | 98 (18.7) | -0.018 |
| Multiple organ failure, No. (%) | 62 (40.0) | 1,150 (23.5) | 0.360 | 62 (40.0) | 203 (38.7) | 0.027 |
| Immunological diseases, No. (%) | 18 (11.6) | 767 (15.7) | 0.119 | 18 (11.6) | 72 (13.7) | -0.063 |
| Surgical operation, No. (%) | 138 (89.0) | 3,024 (61.8) | 0.639 | 138 (89.0) | 459 (87.4) | 0.050 |
| Organ biopsy, No. (%) | 45 (29.0) | 812 (16.6) | 0.300 | 45 (29.0) | 160 (30.5) | -0.032 |
| Pneumonia, No. (%) | 108 (69.7) | 2,648 (54.1) | 0.339 | 108 (69.7) | 356 (67.8) | 0.040 |

Note. PS, propensity score; N-CLABSI, non-central line-associated bloodstream infection; SD, standard deviation; APACHE, Acute Physiology and Chronic Health Evaluation; ICU, intensive care unit; MDRO, multidrug-resistant organism.

^*^ An absolute value ≤ 0.1 indicates a negligible difference in the mean or prevalence of a covariate between groups.

^†^ Matching scale is 1:4, calipers value is 0.02.
