# Supplementary material for: The clinical impacts and risk factors for non-central line-associated bloodstream infection in 5046 intensive care unit patients: an observational study based on electronic medical records
Source: Crit Care. 2019 Feb 18;23:52. doi: 10.1186/s13054-019-2353-5 (PMC6379966; doi:10.1186/s13054-019-2353-5)
Supplement: Supplementary file 3 — Table S3. PS model for mortality. (DOCX 21 kb) [file 13054_2019_2353_MOESM3_ESM.docx]

**Additional file 3**

**Table S3. PS model for mortality**

| Covariates | Before PS matching | | | After PS matching^†^ | | |
| --- | --- | --- | --- | --- | --- | --- |
|  | **N-CLABSI (n=155)** | **Without N-CLABSI (n=4891)** | **Standardized**  **Difference^*^** | **N-CLABSI (n=153)** | **Without N-CLABSI (n=487)** | **Standardized**  **Difference** |
| Age (y), mean ± SD | 53.3 ± 16.4 | 56.9 ± 17.7 | -0.213 | 53.7 ± 16.1 | 54.1 ± 17.9 | -0.025 |
| Sex, No. male (%) | 110 (71.0) | 3,174 (64.9) | 0.130 | 108 (70.6) | 346 (71.0) | -0.010 |
| APACHE II score on ICU admission, mean ± SD | 21.1 ± 8.0 | 19.0 ± 8.1 | 0.249 | 21.0 ± 8.0 | 20.5 ± 7.7 | 0.055 |
| Shock, No. (%) | 69 (44.5) | 1,118 (22.9) | 0.471 | 67 (43.8) | 191 (39.2) | 0.093 |
| Multiple organ failure, No. (%) | 62 (40.0) | 1,150 (23.5) | 0.360 | 60 (39.2) | 168 (34.5) | 0.098 |
| Surgical operation, No. (%) | 138 (89.0) | 3,024 (61.8) | 0.639 | 136 (88.9) | 421 (86.4) | 0.074 |
| Intravascular catheters, No. (%) | 143 (92.3) | 2,962 (60.6) | 0.367 | 141 (92.2) | 438 (89.9) | 0.078 |
| MV, No. (%) | 148 (95.5) | 4,386 (89.7) | 0.255 | 146 (95.4) | 459 (94.3) | 0.053 |
| MDRO, No. (%) | 123 (79.4) | 1,660 (33.9) | 1.031 | 121 (79.1) | 365 (74.9) | 0.098 |

Note. PS, propensity score; N-CLABSI, non-central line-associated bloodstream infection; SD, standard deviation; APACHE, Acute Physiology and Chronic Health Evaluation; ICU, intensive care unit; MV, mechanical ventilation; MDRO, multidrug-resistant organism.

^*^ An absolute value ≤ 0.1 indicates a negligible difference in the mean or prevalence of a covariate between groups.

^†^ Matching scale is 1:4, calipers value is 0.02.
