# Supplementary material for: The clinical impacts and risk factors for non-central line-associated bloodstream infection in 5046 intensive care unit patients: an observational study based on electronic medical records
Source: Crit Care. 2019 Feb 18;23:52. doi: 10.1186/s13054-019-2353-5 (PMC6379966; doi:10.1186/s13054-019-2353-5)
Supplement: Supplementary file 4 — Table S4. Adjusting factors for the clinical impacts of N-CLABSI in the PS-matched cohorts. (DOCX 18 kb) [file 13054_2019_2353_MOESM4_ESM.docx]

**Additional file 4**

**Table S4. Adjusting factors for the clinical impacts of N-CLABSI in the PS-matched cohorts**

| Outcomes | Adjusted β or OR | Adjusting factors |
| --- | --- | --- |
| Logarithmic value of LOS in ICU | β = 0.217 | surgical operation, MDRO, immunological diseases, intravascular catheters, trauma, pancreatitis, pneumonia and HAIs other than NBSI, pneumonia and IAI |
| Logarithmic value of LOS in hospital | β = 0.137 | surgical operation, MDRO, MV, shock, sepsis, pancreatitis and HAIs other than NBSI, pneumonia and IAI |
| Logarithmic value of hospitalization costs | β = 0.213 | male, surgical operation, MDRO, intravascular catheters, immunological diseases, pancreatitis, pneumonia, IAI, organ biopsy and HAIs other than NBSI, pneumonia and IAI |
| Death in ICU | OR = 2.175 | Age, APACHE II score on ICU admission, chronic underlying diseases, multiple organ failure, shock, surgical operation, MDRO, intravascular catheters, pancreatitis, pneumonia, and HAIs other than NBSI, pneumonia and IAI |
| Predicted death in ICU | OR = 2.960 | Age, APACHE II score on ICU admission, chronic underlying diseases, multiple organ failure, shock, ARDS, gastrointestinal bleeding, trauma, MDRO, organ biopsy, intravascular catheters, MV, UC, sepsis, pneumonia and IAI |

NOTE. N-CLABSI, non-central line-associated bloodstream infection; PS, propensity score; β, unstandardized coefficients of a multiple linear regression model; OR: odds ratio of a logistic regression model; LOS, length of stay; ICU, intensive care unit; MDRO, multidrug-resistant organism; HAI, healthcare-associated infection; BSI, bloodstream infection; IAI, intraabdominal infection; MV, mechanical ventilation; APACHE, Acute Physiology and Chronic Health Evaluation; ARDS, acute respiratory distress syndrome; UC, urinary catheter; predicted death in ICU included death in ICU and discharging from ICU against medical advice because of critical conditions and the desire to pass away at home. There was no multicollinearity within the variables in each of the final models.
